# Longitudinal impact of weight change on blood pressure in University students

**DOI:** 10.1038/s41440-025-02370-1

**Published:** 2025-09-11

**Authors:** Nobuko Yamada-Goto, Nahomi Sei, Ayano Murai-Takeda, Tatsuhiko Azegami, Keika Sakakibara-Adachi, Kaori Hayashi, Mikako Inokuchi, Hiroshi Hirose

**Affiliations:** 1 Keio University Health Center, Kanagawa, Japan; 2 Division of Nephrology, Endocrinology, and Metabolism, Department of Internal Medicine, Keio University School of Medicine, Tokyo, Japan; 3 Department of Pediatrics, Keio University School of Medicine, Tokyo, Japan

**Keywords:** hypertension, obesity, weight gain, weight loss, young adults

## Abstract

Obesity-related hypertension is increasing in Japan, but limited evidence exists on how weight change in early adulthood affects blood pressure (BP). We aimed to investigate the longitudinal relationship between weight change and BP among Japanese university students. We analyzed data from 20,929 first-year students who underwent annual health checkups at Keio University from 2013 to 2016 and again three years later. Students were categorized into seven groups by percentage weight change. Associations between weight change and changes in systolic and diastolic BP (ΔSBP/ΔDBP) were examined using sex-stratified analyses and restricted cubic spline (RCS) models. Weight gain was linked to BP increases, while weight loss was associated with BP reductions. In men, weight gain >3% increased SBP and DBP, and weight loss >3% reduced SBP; DBP reduction was evident with >10% weight loss. In women, SBP increased with weight gain >5%, and DBP increased with >10% weight gain; SBP decreased with >10% weight loss. RCS models revealed nonlinear, dose-dependent associations with sex-specific patterns. Absolute BMI was also positively associated with BP, with inflection points consistently observed at BMI 21.6 kg/m² for SBP and DBP in women, and for DBP in men. Even moderate weight changes over three years significantly influenced BP, with SBP being more responsive than DBP and stronger effects observed in men. These findings suggest that even during shorter periods in young adults, body weight change has a high impact on BP and highlight the potential public health benefits of strategies aimed at preventing weight gain throughout adulthood.

**Figure:**
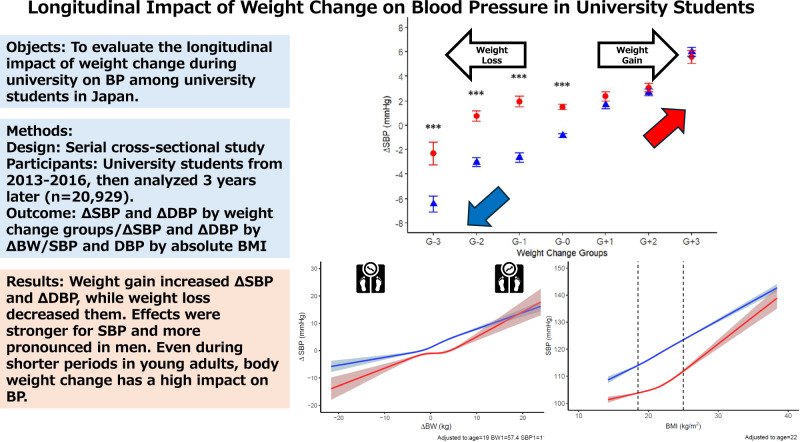
This study examined the longitudinal impact of weight change on blood pressure in university students in Japan. Weight gain increased ΔSBP and ΔDBP, while weight loss decreased them. Effects were stronger for SBP and more pronounced in men. Even during shorter periods in young adults, body weight change has a high impact on BP

This study examined the longitudinal impact of weight change on blood pressure in university students in Japan. Weight gain increased ΔSBP and ΔDBP, while weight loss decreased them. Effects were stronger for SBP and more pronounced in men. Even during shorter periods in young adults, body weight change has a high impact on BP

## Abstract (web summary)

This study examined the longitudinal impact of weight change on blood pressure in university students in Japan. Weight gain increased ΔSBP and ΔDBP, while weight loss decreased them. Effects were stronger for SBP and more pronounced in men. Even during shorter periods in young adults, body weight change has a high impact on BP.

## Introduction

Hypertension is a critical global health concern. Morbidity and mortality risks due to cardiovascular disease (CVD) increase with elevations in blood pressure (BP) > 120/80 mmHg, as observed in Japan and other countries [1]. Although hypertension is usually more common among elderly individuals, recent epidemiological studies have shown that it may start during adolescence and persist into adulthood [2]. Furthermore, the earlier the hypertension onset, the higher the CVD risk and mortality [2]. A systematic review and meta-analysis of young adults aged 18–45 with high BP, which contained data from Europe, the U.S., and Asia, including Japan, has revealed continuous and graded associations between categorical BP increments and increased risks of cardiovascular events, coronary heart disease, stroke, and all-cause mortality [3]. In a study in which Japanese university students were re-examined after 8–26 years, hypertension was observed in 44.6% of the hypertensive group but only in 9.2% of the normotensive group [4]. Therefore, understanding the current trends of hypertension among Japanese university students to prevent future hypertension and CVD is crucial.

BP increases with increasing body weight [5]. The prevalence of obesity-related hypertension has increased in Japan [6]. This relationship has been demonstrated in several cohort studies in Japan [7–9] and overseas [10–17]. Hypertension incidence is higher in individuals with greater weight gain from youth to middle/advanced age [6, 10]. Weight gain of ≥2 kg for 4 years was strongly related to an increased risk for hypertension in middle-aged men [7], and weight gain ≥2 kg for 16 years from young adults was strongly related to an increased hypertension risk in middle-aged women [10]. Conversely, a 1.0 kg weight reduction has been estimated to lower systolic BP (SBP) by approximately 1.1 mmHg and diastolic BP (DBP) by approximately 0.9 mmHg in a meta-analysis [18]. Another meta-analysis revealed a significant reduction in BP (ΔSBP, −4.5; ΔDBP, −3.2 mmHg) following a 4-kg weight reduction [19]. Furthermore, a study found a significant reduction in BP with a 3% or more weight reduction in obese Japanese individuals [20].

We previously reported that Japanese university students with high BP had higher body weights during junior high school [21]. This study aimed to assess the effect of weight change on BP in university students, which would contribute to the evidence on CVD prevention.

## Methods

### Study population and data sources

This study complied with the Declaration of Helsinki and was approved by the Research Ethics Review Committee of the Keio University (approval number 24-001). Informed consent was obtained using opt-out methods according to the local guidelines of the Ethics Committee. This retrospective cohort study included asymptomatic Japanese university students (men and women) who attended annual health checkups at the Keio University Health Center. We included newly enrolled 1st year students from 2013 to 2016 who were under 25 years of age and participated in the annual health check-up 3 years later given that the primary objective was to evaluate the BP of young individuals. The height, weight, and BP of all students were measured. We assessed the effects of changes in body weight on differences in BP during this period.

### Data collection and definitions of BP, BMI categories, and body weight change groups

A trained nurse measured the BP using an electronic sphygmomanometer (BP-103i II; Omron Colin Co., Ltd., Tokyo, Japan) placed on the right arm of the study participants in a relaxed sitting position. A 12 cm-wide cuff with an arm circumference of 22–32 cm was utilized. BP was re-measured if it was ≥140/90 mmHg, following the institution’s health checkup manual. The measured BP was categorized according to the cut-off points for office BP stated in the Japanese Society of Hypertension Guidelines for the Management of Hypertension (JSH 2019) [6]: normal BP (SBP < 120 and DBP < 80 mmHg), high normal BP (SBP: 120–129 and DBP < 80 mmHg), elevated BP (SBP: 130–139 and/or DBP: 80–89 mmHg), Grade I hypertension (SBP: 140–159 and/or DBP: 90–99 mmHg), Grade II hypertension (SBP: 160–179 and/or DBP 100–109 mmHg), Grade III hypertension (SBP $\ge \,$180 and/or DBP $\ge \,$11  mmHg), and isolated systolic hypertension (SBP $\ge \,$140 and DBP < 90 mmHg). Additionally, BP values were categorized into two groups: normal BP defined as SBP < 120 mmHg and DBP < 80 mmHg, and high BP, defined as SBP ≥ 120 mmHg or DBP ≥ 80 mmHg. The standing height and body weight were measured without shoes or clothing.

BMI was calculated as the body weight divided by the square of the height (kg/m^2^) and categorized as normal weight (BMI 18.5 to <5 kg/m^2^), obese (BMI ≥ 25 kg/m^2^), or underweight (BMI < 18.5 kg/m^2^) according to the Guidelines for the Management of Obese Disease 2022 published by the Japan Society for the Study of Obesity [22].

To evaluate the impact of body weight change over 3 years on the amount of change in SBP (ΔSBP) and DBP (ΔDBP), we divided students into seven groups: weight loss of >10% (G-3), weight loss of >5% to ≤10% (G-2), weight loss of >3% to ≤5% (G-1), weight change form ≥−3% to ≤ +3% (G-0), weight gain of >3% to ≤5% (G + 1), weight gain of >5% to ≤10% (G + 2), and weight gain of >10% (G + 3). The specific thresholds of 3%, 5%, and 10% used in this study were based on Japanese data and aligned with the weight loss targets recommended in the Guidelines for the Management of Obesity Disease 2022 [22]. Although the guideline addresses only weight loss, we applied the same thresholds symmetrically to categorize weight gain for comparative analysis.

### Statistical analysis

Data processing and statistical analyses were conducted using SPSS Statistics version 29.0 (IBM Corp., Armonk, NY, USA) and R version 4.5.0 with RStudio (R Foundation for Statistical Computing, Vienna, Austria). All variables were summarized using descriptive statistics and presented as means ± standard deviations (SD). Group comparisons were performed using SPSS. Categorical variables were compared using chi-square tests for independence, and continuous variables were analyzed across body weight change groups using one-way analysis of variance (ANOVA). Post-hoc comparisons were performed using Tukey’s test when the assumption of equal variances was satisfied, and the Games–Howell test when it was not. A two-sided p-value of <0.05 was considered statistically significant.

To investigate the association between continuous changes in body weight (ΔBW) and ΔSBP/ΔDBP, we employed sex-stratified restricted cubic spline (RCS) regression models in R, adjusting for age, baseline body weight, and baseline SBP or DBP. Interaction terms between sex and ΔBW were included to assess sex-specific associations. Visualizations of post-hoc comparisons and spline models were generated using base R and the ggplot2 package.

To enhance the practical applicability of our findings in student health guidance, we further examined the associations between absolute BMI (at baseline and follow-up) and SBP/DBP using sex-stratified RCS models adjusted for age. Interaction terms for sex were included, and to better interpret sex differences in the strength and direction of these associations, we calculated the first derivatives (i.e., slopes) of the predicted spline functions for men and women separately.

RCS models were constructed with four knots placed at the 5th, 35th, 65th, and 95th percentiles of ΔBW or ΔBMI, as recommended by Harrell (Regression Modeling Strategies, 2nd edition) [23], allowing flexible modeling of potential non-linear associations while minimizing the risk of overfitting.

Details of the statistical methods used for graphical representations are also provided in the respective figure legends.

## Results

### Baseline characteristics of the students

**Fig. 1:**
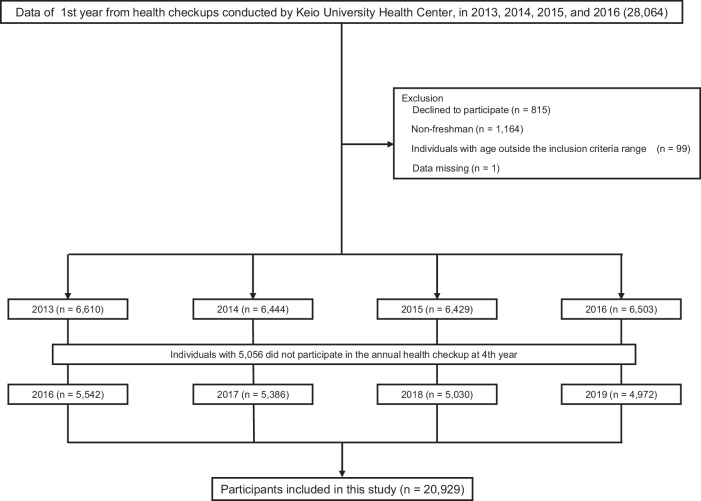
Flowchart showing the process of participant selection. We extracted data from 28,064 individuals enrolled in health checkups at Keio University conducted by the Keio University Health Center, Japan, between 2013 and 2016. After excluding 7135 individuals (815 individuals who declined to participate in this study, 1164 individuals in the 1st year, but not new students, 99 with age outside the inclusion criteria range, 1 with missing blood pressure data, and 5056 who did not participate again in the annual health checkup 3 years later) and 20,929 individuals were included in the analysis

Of the 28,064 individuals, 7135 were excluded (815 individuals who declined to participate, 1164 individuals in the 1st year, but not new students, 99 with age outside the inclusion criteria range, 1 with missing BP data, and 5056 who did not participate in the annual health checkup 3 years later) (Fig. 1). To assess the potential for selection bias, we compared the included and excluded participants (Supplementary Table 1). Although there were statistically significant differences in age and height differed significantly, the differences were minimal. No significant differences were observed in weight, BMI, BMI categories, BP categories, the prevalence of isolated systolic hypertension, or the proportion with BP ≥ 120/80 mmHg. Therefore, we consider the risk of substantial selection bias to be low.

Baseline and 3 years follow-up comparisons of anthropometric and BP measurements in men and women are shown in Table 1. From the 1st to 4th year, both sexes exhibited significant increases in height, weight, BMI, SBP, and DBP, with men consistently exhibiting higher values than women across all measures. Despite these sex differences, both groups showed similar upward trends over time.

**Table 1:** Characteristics of the students included in the study

	Men (*n* = 13,061)			Women (*n* = 7868)		
	1st year	4th year	*p* value	1st year	4th year	*p* value
Age, year	19.4 ± 0.6	22.4 ± 0.6	< 0.001	19.2 ± 0.5	22.2 ± 0.5	< 0.001
Height, cm	171.9 ± 5.7	172.4 ± 5.7	< 0.001	158.9 ± 5.3	159.3 ± 5.3	< 0.001
Weight, kg	63.1 ± 9.2	64.3 ± 9.8	< 0.001	50.4 ± 6.3	51.0 ± 6.5	< 0.001
BMI, kg/m^2^	21.3 ± 2.8	21.6 ± 3.0	< 0.001	19.9 ± 2.2	20.1 ± 2.2	< 0.001
SBP, mmHg	117.9 ± 12.3	118.2 ± 12.6	< 0.001	103.9 ± 11.3	103.9 ± 11.3	< 0.001
DBP, mmHg	63.9 ± 8.0	65.7 ± 8.2	< 0.001	59.3 ± 7.4	60.8 ± 7.6	< 0.001
BMI category (JASSO-2022), *n* (%)						
Underweight	1631 (12.5)	1,465 (11.2)	< 0.001	1930 (24.5)	1830 (23.3)	0.02
Normal	10,238 (78.4)	10,136 (77.6)		5756 (73.2)	5827 (74.1)	
Obese	1192 (9.1)	1460 (11.2)		182 (2.3)	211 (2.7)	
BP category (JSH-2019), *n* (%)						
Normal BP	7215 (55.2)	7298 (55.9)	0.66	7149 (90.9)	6847 (87.0)	0.32
High normal BP	3343 (25.6)	3188 (24.4)		522 (6.6)	752 (9.6)	
Elevated BP	2202 (16.9)	2140 (16.4)		180 (2.3)	248 (3.2)	
Grade I hypertension	280 (2.1)	385 (2.9)		15 (0.2)	18 (0.2)	
Grade II hypertension	19 (0.1)	47 (0.4)		1 (0.0)	3 (0.0)	
Grade III hypertension	2 (0.0)	3 (0.0)		1 (0.0)	0 (0.0)	
Isolated systolic hypertension	282 (2.2)	377 (2.9)	< 0.001	13 (0.2)	12 (0.2)	0.8
*n* (%) of 120/80 mmHg or higher	5846 (44.8)	5763 (44.1)	0.22	719 (9.1)	1021 (13.0)	< 0.001

Data are presented as mean ± standard deviation (SD), or number (percentage). Chi-square independence tests were used to compare the categorical variables

Interaction between sex and ΔBW was modeled to allow for sex-specific associations

The following covariates were included in the multivariable spline models: age, baseline BW, and baseline SBP for ΔSBP models; age, baseline BW, and baseline DBP for ΔDBP models. Interaction between sex and ΔBW was modeled to allow for sex-specific associations

*BMI* Body mass index, *SBP* Systolic blood pressure, *DBP* Diastolic blood pressure, *BP* Blood pressure, *JASSO* Japan Society for the Study of Obesity, *JSH* The Japanese Society of Hypertension

In both years, the proportion of individuals with a normal BMI was higher in men than in women. Men also had fewer underweight individuals and more classified as obese. Over time, the proportions of underweight and normal-weight individuals declined, while the proportion of obese men rose. Among women, the proportion of underweight individuals declined, while both the normal-weight and obese categories increased by the 4th year.

The prevalence of isolated systolic hypertension significantly increased in men, with no other significant shifts in BP classification in either sex. The proportion of men with high BP was already elevated at 44.8% in the 1st year and remained unchanged in the 4th year. In contrast, the proportion of women with high BP rose considerably from 9.1% to 13.0% over the same period.

### Baseline characteristics of weight change groups

Table 2a presents the characteristics of body weight change groups among men. Compared with the G-0 group, the G-3 and G-2 groups had significantly higher age, BMI, SBP, and DBP at baseline, while the G-1 group had significantly higher BMI at baseline. At the 4th year follow-up, the G-3 group had significantly higher age, SBP, and DBP; the G-2 group had significantly higher age and BMI; and the G-1 group continued to show significantly higher BMI than the G-0 group. Among the weight gain groups, G + 1, G + 2, and G + 3 groups showed no significant differences from the G-0 group at baseline. However, at the 4th year follow-up, these groups had significantly higher BMI, SBP, and DBP compared with the G-0 group.

**Table 2:** a. Characteristics of body weight change groups divided according to the percentage body weight change from baseline to the end of the follow-up (men). b. Characteristics of body weight change groups divided according to the percentage body weight change from baseline to the end of the follow-up (women)

	Groups	Percent of BW change during follow up (%)		Age, year	*p* value to G-0	BMI, kg/m^2^	*p* value to G-0	SBP, mmHg	*p* value to G-0	DBP, mmHg	*p* value to G-0
BW loss	G-3	> −10	1st year	19.5 ± 0.7	<0.001	24.9 ± 3.5	<0.001	125.4 ± 12.3	<0.001	67.3 ± 8.3	<0.001
	(*n* = 414)		4th year	22.5 ± 0.7	<0.001	21.3 ± 2.7	0.332	119.0 ± 13.1	0.006	66.5 ± 8.3	0.013
			^a^*p* value	<0.001		<0.001		<0.001		0.054	
	G-2	> −5 to −10	1st year	19.4 ± 0.7	<0.001	22.4 ± 2.8	<0.001	119.8 ± 12.7	<0.001	64.7 ± 8.1	0.003
	(*n* = 1199)		4th year	22.4 ± 0.7	<0.001	20.7 ± 2.5	0.026	116.7 ± 12.7	1.000	65.4 ± 8.3	0.878
			^a^*p* value	<0.001		<0.001		<0.001		0.005	
	G-1	> −3 to −5	1st year	19.4 ± 0.7	0.748	21.6 ± 2.6	<0.001	118.3 ± 12.5	0.301	63.9 ± 8.0	0.974
	(n = 1067)		4th year	22.4 ± 0.7	0.748	20.6 ± 2.5	0.002	115.7 ± 12.2	0.295	65.0 ± 8.0	1.000
			^a^*p* value	<0.001		<0.001		<0.001		<0.001	
Control	G0	> −3 to +3	1st year	19.3 ± 0.6	-	21.0 ± 2.6	-	117.4 ± 12.1	-	63.7 ± 7.8	-
	(n = 5124)		4th year	22.3 ± 0.6	-	21.0 ± 2.6	-	116.6 ± 12.0	-	65.0 ± 7.9	-
			^a^*p* value	<0.001		<0.001		<0.001		<0.001	
BW gain	G + 1	> +3 to +5	1st year	19.3 ± 0.6	0.973	20.8 ± 2.5	0.091	116.7 ± 11.9	0.369	63.2 ± 8.0	0.384
	(*n* = 1551)		4th year	22.3 ± 0.6	0.973	21.6 ± 2.6	<0.001	118.4 ± 12.2	<0.001	65.8 ± 8.1	0.014
			^a^*p* value	<0.001		<0.001		<0.001		<0.001	
	G + 2	> +5 to +10	1st year	19.3 ± 0.7	0.053	21.0 ± 2.6	1.000	117.4 ± 12.2	1.000	63.6 ± 8.0	0.999
	(*n* = 2365)		4th year	22.4 ± 0.7	0.053	22.4 ± 2.9	<0.001	120.1 ± 12.6	<0.001	66.4 ± 8.2	<0.001
			^a^*p* value	<0.001		<0.001		<0.001		<0.001	
	G + 3	> +10	1st year	19.4 ± 0.7	0.778	21.3 ± 2.8	0.088	117.7 ± 12.3	0.997	63.8 ± 8.0	1.000
	(*n* = 1341)		4th year	22.4 ± 0.7	0.778	24.3 ± 3.5	<0.001	123.6 ± 13.0	<0.001	67.7 ± 8.6	<0.001
			^a^*p* value	<0.001		<0.001		<0.001		<0.001	
BW loss	G-3	> -10	1st year	19.2 ± 0.6	0.869	22.0 ± 2.7	<0.001	107.0 ± 12.5	0.039	60.6 ± 8.2	0.288
	(*n* = 215)		4th year	22.2 ± 0.6	0.869	19.0 ± 2.3	<0.001	104.6 ± 13.1	0.871	60.7 ± 8.2	1.000
			^a^*p* value	<0.001		<0.001		0.016		0.959	
	G-2	> −5 to −10	1st year	19.2 ± 0.5	0.917	20.9 ± 2.2	<0.001	105.1 ± 11.9	0.473	59.5 ± 7.8	1.000
	(*n* = 848)		4th year	22.2 ± 0.5	0.917	19.3 ± 2.0	<0.001	105.9 ± 11.7	1.000	61.1 ± 7.6	0.814
			^a^*p* value	<0.001		<0.001		0.078		<0.001	
	G-1	> −3 to −5	1st year	19.2 ± 0.5	0.999	20.3 ± 2.1	<0.001	103.7 ± 10.4	0.789	59.1 ± 7.1	0.869
	(n = 685)		4th year	22.2 ± 0.5	0.999	19.4 ± 2.0	<0.001	105.6 ± 11.4	1.000	61.0 ± 7.5	0.979
			^a^*p* value	<0.001		<0.001		<0.001		<0.001	
Control	G0	> −3 to +3	1st year	19.2 ± 0.5	-	19.9 ± 2.0		104.3 ± 11.1	-	59.5 ± 7.4	-
	(*n* = 3236)		4th year	22.2 ± 0.5	-	19.8 ± 2.0		105.8 ± 11.4	-	60.7 ± 7.5	-
			^a^*p* value	<0.001		<0.001		<0.001		<0.001	
BW gain	G + 1	> +3 to +5	1st year	19.2 ± 0.5	1.000	19.6 ± 2.0	<0.001	103.3 ± 11.3	0.246	59.2 ± 7.6	0.975
	(*n* = 982)		4th year	22.2 ± 0.5	1.000	20.3 ± 2.0	<0.001	105.7 ± 11.3	1.000	60.5 ± 7.7	0.991
			^a^*p* value	<0.001		<0.001		<0.001		<0.001	
	G + 2	> +5 to +10	1st year	19.2 ± 0.5	0.565	19.5 ± 2.1	<0.001	102.7 ± 11.1	<0.001	58.7 ± 7.2	0.036
	(*n* = 1287)		4th year	22.2 ± 0.5	0.565	20.7 ± 2.3	<0.001	105.7 ± 11.5	1.000	60.5 ± 7.3	0.932
			^a^*p* value	<0.001		<0.001		<0.001		<0.001	
	G + 3	>+10	1st year	19.2 ± 0.6	0.194	19.4 ± 2.4	<0.001	102.9 ± 11.7	0.097	58.9 ± 7.6	0.621
	(*n* = 615)		4th year	22.2 ± 0.6	0.194	21.9 ± 2.8	<0.001	108.5 ± 12.6	<0.001	61.9 ± 8.1	0.008
			^a^*p* value	<0.001		<0.001		<0.001		<0.001	

All variables were statistically described, and measurements were reported as mean ± standard deviation (SD)

One-way analysis of variance was conducted, followed by the Tukey’s test for equal variances

The Games–Howell test for unequal variances was used for post-hoc analysis to compare the weight change groups

Statistical significance was set at *p* < 0.05 using two-sided tests

^a^p-value indicates whether the change in the 4th year is statistically significant compared to the 1st year

Table 2b presents the characteristics of body weight change groups among women. At the baseline, the G-3 group had significantly higher BMI and SBP, while the G-2 and G-1 groups had significantly higher BMI compared with the G-0 group. By the 4th year follow-up, the G-3, G-2, and G-1 groups had significantly lower BMI than the G-0 group.

Among the weight gain groups, G + 1 and G + 3 had significantly lower BMI at baseline, and G + 2 group had significantly lower BMI, SBP, and DBP compared with the G-0 group. At the 4th year follow-up, the G + 1 and G + 2 groups had higher BMI, while the G + 3 group had higher BMI, SBP, and DBP than the G-0 group.

### ΔSBP and ΔDBP in weight change groups

**Fig. 2:**
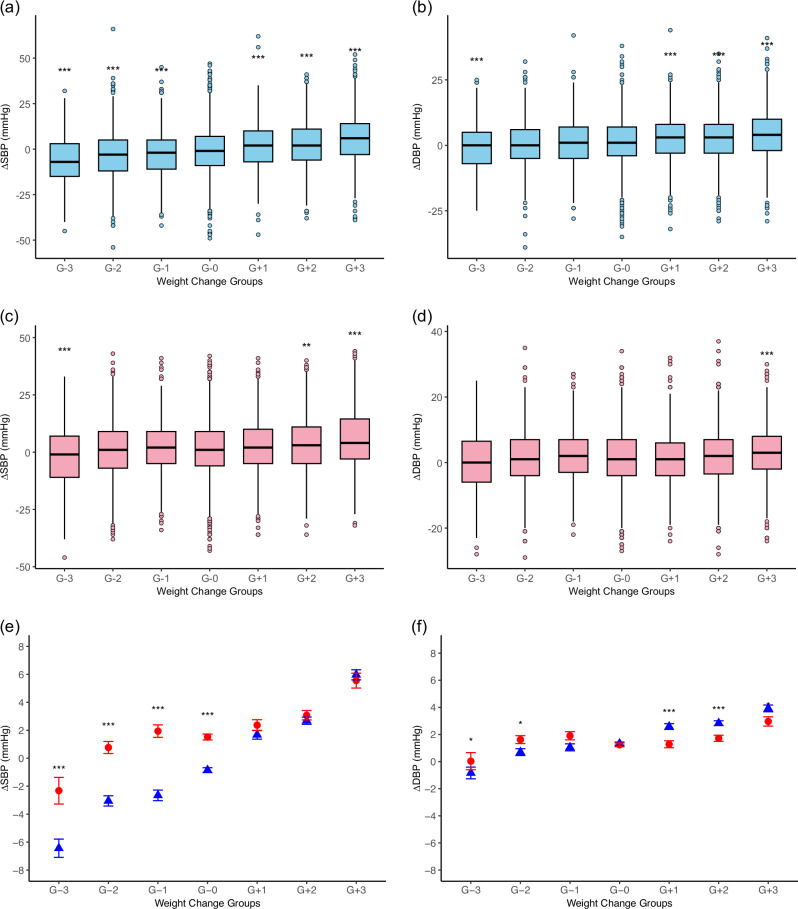
ΔSBP and ΔDBP in weight change groups. Data are presented as boxplots with median, interquartile range (IQR), and outliers. G-3: weight loss >10%; G-2: weight loss between 5 and 10%; G-1: weight loss between 3 and 5%; G-0: weight change between −3% and +3% (reference group); G + 1: weight gain between 3 and 5%; G + 2: weight gain between 5 and 10%; G + 3: weight gain >10%. Panels show: **a** ΔSBP in men, **b** ΔDBP in men, **c** ΔSBP in women, and **d** ΔDBP in women, based on % changes in body weight from baseline to the end of follow-up. A one-way analysis of variance followed by Tukey’s post-hoc test was conducted to compare the weight change groups. **p < 0.01 and ***p < 0.001 indicate statistical significance compared with the G-0 group. Mean ΔSBP (**e**) and ΔDBP (**f**) by sex (men: blue triangles, women: red circles) across weight change groups. Error bars represent standard errors of the mean (±SE). Within each category, sex differences were tested by two-sample t-test. *p < 0.05, **p < 0.01, and ***p < 0.001 indicates a significant difference between sexes

In the analysis of the sex-specific weight change groups presented in Table 2, men in all three weight loss groups showed significant reductions in ΔSBP compared with the control group (G-0), while all three weight gain groups showed significant increases in ΔSBP (Fig. 2a). For ΔDBP in men, significant changes were observed in the G-3, G + 1, G + 2, and G + 3 groups (Fig. 2b). Among women, significant changes in ΔSBP were observed in the G-3, G + 2, and G + 3 groups (Fig. 2c), while ΔDBP showed a significant change only in the G + 3 group (Fig. 2d).

To further examine sex differences in the effect of weight change groups on BP, ΔSBP and ΔDBP were compared between men and women on the same graphs (Fig. 2e, f). For ΔSBP, both sexes showed greater reductions with increasing weight loss, though the effect was more pronounced in men. For ΔDBP, overall changes were minimal across groups. However, statistically significant sex differences were observed: women showed higher ΔDBP than men in the moderate weight loss groups (G-2 and G-1), whereas men showed higher ΔDBP than women in the weight gain groups (G + 1 to G + 3).

#### Association between ΔBW and ΔSBP/ΔDBP by sex

**Fig. 3:**
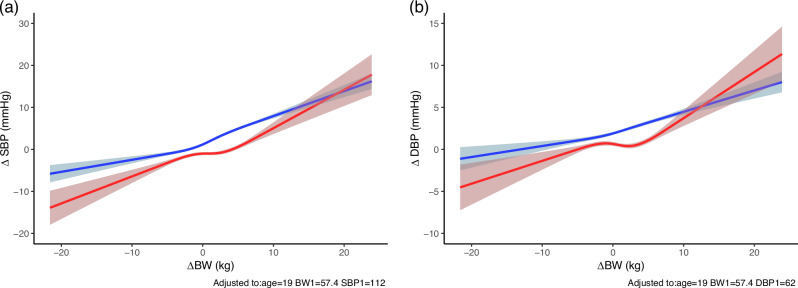
The association of ΔBW and ΔSBP/ΔDBP by sex. RCS models including an interaction between ΔBW and sex, adjusted for age, baseline BW, and baseline SBP or DBP, **a** ΔSBP, and **b** ΔDBP. Solid lines represent predicted values; shaded areas represent 95% CIs. Predictions were calculated at age 19, BW = 57.4 kg, SBP = 112 mmHg, and DBP = 62 mmHg. Results are shown separately for men (blue) and women (red). Models were fitted using R (v4.5.0) with the rms and ggplot2 packages

Consistent with the categorical analyses, greater weight loss was associated with larger reductions in ΔSBP, particularly among women (Fig. 3a). In men, the relationship between ΔBW and ΔSBP was nearly linear, with weight gain producing a more pronounced increase in ΔSBP than the BP-lowering effect observed with weight loss. In women, ΔSBP remained relatively stable within the narrow ΔBW range of 0 to +2 kg, but outside this range—both with weight loss and greater weight gain—a roughly linear association emerged.

Figure 3b shows similar patterns for ΔDBP, although with gentler slopes. In men, the relationship remained approximately linear across the ΔBW range. In women, ΔDBP also showed minimal change near 0 to +2 kg ΔBW, with more apparent increases or decreases outside that range. Pearson correlation coefficients reflected these trends but underestimated the observed associations due to their assumption of linearity. In men, ΔBW was weakly correlated with ΔSBP (*r* = 0.229) and ΔDBP (*r* = 0.135); in women, correlations were negligible (*r* = 0.121 for ΔSBP, *r* = 0.0595 for ΔDBP).

#### Association between absolute BMI and SBP/DBP by sex

**Fig. 4:**
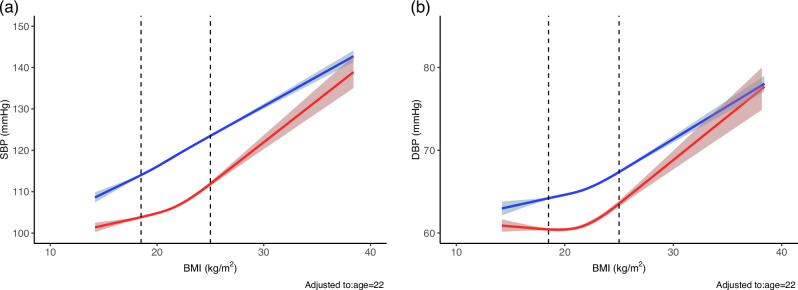
The association of absolute BMI and SBP/DBP by sex. RCS models including an interaction between BMI and sex, adjusted for age, and baseline SBP or DBP, **a** SBP, and **b** DBP. Solid lines represent predicted values; shaded areas represent 95% CIs. Predictions were calculated at age 22. Vertical dashed lines at BMI 18.5 and 25.0 kg/m² represent the normal BMI range (JASSO, 2022). Results are shown separately for men (blue) and women (red). Models were fitted using R (v4.5.0) with the rms and ggplot2 packages

For both SBP and DBP, women consistently showed lower predicted BP values than men at the same BMI level (Fig. 4a, b). First derivatives of the predicted spline functions with respect to BMI were estimated separately for men and women using finite differences to approximate instantaneous slopes across the BMI range. We then computed the pointwise differences in these slopes between sexes.

To identify regions of maximal curvature in the BMI–BP relationship, we approximated the second derivatives—reflecting the rate of change in slope—and determined the BMI values at which the absolute rate of slope change was greatest. These inflection points indicate where the association between BMI and BP shifted most sharply. Notably, the inflection points were consistently occurred at a BMI of 21.6 kg/m² for female SBP, female DBP, and male DBP, highlighting a common transitional range in the nonlinear association between BMI and BP. For reference, Pearson correlation coefficients between absolute BMI and SBP/DBP were calculated separately by sex. Among men, the correlation was (*r* = 0.337) for SBP and (*r* = 0.202) for DBP. Among women, the correlation values were *r* = 0.206 for SBP and *r* = 0.102 for DBP. These results suggest a moderate linear association for SBP, particularly in men, and weaker associations for DBP, consistent with the nonlinear patterns observed in the spline models.

## Discussion

This three-year longitudinal study of Japanese university students demonstrated that changes in body weight were significantly associated with changes in BP, with notable sex differences and a nonlinear relationship. In men, weight gain had a more pronounced impact on SBP, suggesting that even modest increases may elevate SBP. In women, noticeable changes in BP were less likely to occur unless weight change exceeded a certain threshold, after which the impact increased sharply, particularly once a specific BMI threshold was crossed.

ΔSBP and ΔDBP over 3 years depended on the change in body weight. The greater the weight gain percentage, the greater the BP increase; the greater the weight loss percentage, the greater the BP decrease. Overall, weight change had a stronger effect on ΔSBP than on ΔDBP, and this influence was more pronounced in men than in women.

The analysis of changes in ΔBW and ΔSBP/ΔDBP revealed sex-specific and nonlinear associations, with SBP being more sensitive to weight change than DBP. In men, ΔSBP increased almost linearly with weight gain, whereas weight loss produced a smaller reduction, suggesting a stronger vascular response to weight gain than to weight loss. In women, SBP remained stable with small weight changes (0 to +2 kg), indicating a threshold beyond which both weight gain and weight loss led to more noticeable BP shifts. DBP showed similar but more modest patterns, with clearer associations emerging only with larger weight changes.

The analysis of absolute BMI and SBP/DBP showed that women had consistently lower predicted BP than men at any given BMI, particularly in the lower to mid-BMI ranges. While the BMI–BP relationship was gradual below 25 kg/m² in both sexes, it became steeper in the overweight range. In men, SBP rose steadily with increasing BMI, whereas in women, SBP remained stable until a threshold—around 21.6 kg/m²—after which it rose sharply. This inflection point was also observed in DBP for both sexes, suggesting a physiological threshold beyond which BP responses to adiposity intensify. These findings highlight the importance of monitoring BMI even within the normal range, particularly in women, due to the risk of early BP elevation.

These findings indicate that even over short periods in young adults, BP changes in parallel with weight changes, consistent with patterns observed in middle-aged populations. BP increases in parallel with the increase in body weight in middle-aged men and women [6, 7, 10]. Conversely, BP decreases in parallel with a decrease in body weight in middle-aged men and women, with most of their BMI being >25 kg/m^2^ [18–20].

Although the mean BMI in our study was <25 kg/m^2^, reduction in ΔSBP was observed in both the 35% to <10% and ≥10% groups, whereas a substantial reduction in ΔDBP was seen only in the ≥10% weight loss group among men. In women, a significant ΔSBP reduction was also observed in the ≥10% weigh loss group, while no significant ΔDBP reduction was detected. Higher BP and BMI were observed at baseline in the groups with greater weight loss, which seemed to be counterintuitive. No differences in the baseline BP and only a slight decrease in BMI at baseline were observed in the group with greater weight gain. A previous meta-analysis of randomized control trials revealed substantially larger BP reductions with an average weight loss of >5 kg than in populations with less weight loss, although the initial BMI (<30 vs ≥30 kg/m^2^) did not markedly influence BP response to weight loss [18]. In the present study, two possible explanations exist for the relatively high baseline BP and BMI in the group with greater weight loss. First, these students may have experienced temporary weight gain during pre-admission exam preparation, which later improved due to healthier routines adopted after entering university. Second, the elevated BP and BMI at baseline may have motivated these students to intentionally adopt healthier habits to reduce their weight.

The Framingham Study revealed that visceral fat volume was more positively correlated with BP and hypertension prevalence than subcutaneous fat volume [24]. Although Japanese individuals typically have a smaller body size than Caucasians, they accumulate visceral fat more easily. In individuals with the same BMI, the incidence of diabetes and hypertension is higher in Asians than in Caucasians [25]. Weight gain between early adulthood and midlife is linked to higher mid-life BP [7, 10]. We previously demonstrated that height, weight, BMI, and overweight status in childhood were positively related to high BP in young adulthood [21]. In the present study, certain mechanisms of hypertension leading to increased body weight may be due to hypertension development caused by visceral fat accumulation.

High BP prevalence in this study was 44.8% in men and 9.1% in women, even in the 1st year. This prevalence is consistent with previous reports [21, 26, 27]. The percentages of women with high BP, as well as mean SBP and DBP in both sexes, increased substantially by the 4th year compared to the 1st year. The annual number of cardiovascular deaths due to hypertension in Japan is estimated to be 100,000, accounting for the largest proportion of all cardiovascular deaths [6]. Approximately 50% of CVD-related deaths are estimated to be caused by BP > 120/80 mm Hg [6]. SBP and DBP independently influenced cardiovascular outcomes in young adults [2, 3]. In the present study, alterations in ΔSBP in the weight-change groups were much greater than those in the ΔDBP group. Among the BP parameters, SBP strongly predicts the risk of CVD [28]. However, its predictive ability was weaker for DBP [29].

In the Suita study, compared with normal BP (SBP  < 120 mmHg and DBP  < 80 mmHg), increased BP (SBP 130–139 mmHg and/or DBP 80–89 mmHg) and hypertension (SBP  ≥  140 and/or DBP  ≥  90 mmHg) were related to a higher risk of CVD, stroke, and coronary heart disease (CHD). High-normal BP (SBP 120–129 mmHg and DBP  < 80 mmHg) is associated with a higher CVD and CHD risk [30]. Therefore, a substantial increase in high normal BP of men in this study will impact future CVD, stroke, and CHD risk in this population.

According to the Framingham Heart Study, compared to the group without hypertension, the odds ratios for the group that experienced hypertension onset at age <35 years were far higher for left ventricular hypertrophy, coronary artery calcification, albuminuria, and diastolic dysfunction [31]. This phenomenon was not observed in the group with onset of hypertension at age ≥45 years [31]. The pathophysiological basis of high BP in young adults and older people is thought to differ. White-coat hypertension, a hyperadrenergic state, a higher secondary hypertension prevalence, and hypertension caused by peripheral BP amplification are more commonly observed in young adults [3]. In young adults with fewer comorbidities or risk factors, the role of increased BP is dominant. Despite the relatively low absolute risk, the difference in absolute risk and the fact that sustained hypertension for longer durations is related to a higher CVD risk imply that early BP management among young adults may result in considerable public health benefits by reducing the CVD risk later in life. Antihypertensive medication in middle-aged and older adults does not completely mitigate the higher CVD risk among those with high BP compared to their counterparts who maintain normal untreated BP during young adulthood [32]. Therefore, properly diagnosing and treating hypertension as well as providing health education on hypertension prevention while still being a university student are extremely crucial.

In our analysis, the associations between body weight and BP—whether examined by weight change categories, ΔBW and ΔSBP/ΔDBP, or absolute BMI and BP—differed by sex. A notable inflection point around BMI 21.6 kg/m^2^ emerged, marking a potential physiological threshold where BP responses to BMI shift sharply—especially in women. These sex-specific differences in hypertension may reflect distinct biological mechanisms. Estrogen promotes vasodilation via nitric oxide production and suppression of angiotensin II receptor type 1, while testosterone may enhance vasoconstriction and renal injury through activation of the renin–angiotensin–aldosterone system [33]. Additionally, differences in fat distribution, low-grade inflammation, renal sodium handling, and regulation of the sympathetic nervous and immune systems contribute to sex-related variation in BP regulation [33]. Regarding the impact of weight gain, increases in SBP were more pronounced than those in DBP, especially among men. This suggests that young men may be more susceptible to SBP elevation with modest weight gain, underscoring the importance of early lifestyle interventions. In contrast, DBP tended to rise more substantially in women, particularly in the context of greater weight gain. Given that DBP is also an independent cardiovascular risk factor, attention to weight management is equally important for women. While prior studies in middle-aged populations have consistently shown stronger associations between SBP and weight gain, evidence in young adults has been limited. The present study helps to fill this gap by demonstrating that such associations are already evident in early adulthood. Importantly, our findings highlight sex-specific trajectories and the existence of a possible threshold BMI, which may inform tailored strategies for cardiovascular risk prevention in younger populations.

Our study had several limitations. First, the likelihood of white-coat hypertension cannot be ruled out because measuring home BP was not possible. Second, we only assessed an extremely short period (3 years) and thus could not evaluate future outcomes such as CVD. Third, our study population comprised urban university students, who may not be representative of the Japanese population. Fourth, racial differences were not considered in this study. Fifth, in this study, we did not assess the impact of confounding factors such as high-calorie diet, excessive salt intake, lack of physical activity, stress, and insufficient sleep on weight gain and hypertension in our study. Although lifestyle questionnaires were administered, they only covered to a subset of students, limiting our ability to conduct a comprehensive analysis. Finally, secondary hypertension could not be ruled out.

In conclusion, our three-year follow-up of university students revealed that changes in SBP and DBP were significantly associated with changes in body weight. Both group-based and continuous variable analyses demonstrated that weight gain was positively associated with increases in SBP and DBP. Similarly, BMI at follow-up was significantly associated with both SBP and DBP. These findings highlight the potentially significant public health impact of strategies to prevent weight gain throughout from early adulthood. Thus, lifestyle interventions to control weight during university may aid in preventing hypertension onset and severity and reducing medical costs. Since 2019, the institutional health checkup system has been improved to systematically collect lifestyle questionnaires with student ID numbers from all students. This enhancement will enable more comprehensive, individualized analyses in future studies, further advancing understanding of lifestyle–BP relationships in young adults.

## Supplementary information

Supplementary Table 1
